# Thrombosis Is Reduced by Inhibition of COX-1, but Unaffected by Inhibition of COX-2, in an Acute Model of Platelet Activation in the Mouse

**DOI:** 10.1371/journal.pone.0020062

**Published:** 2011-05-23

**Authors:** Paul C. Armstrong, Nicholas S. Kirkby, Zetty N. Zain, Michael Emerson, Jane A. Mitchell, Timothy D. Warner

**Affiliations:** 1 The William Harvey Research Institute, Barts and the London School of Medicine and Dentistry, Queen Mary University of London, London, United Kingdom; 2 Cardiothoracic and Stem Cell Pharmacology, National Heart and Lung Institute, Imperial College, London, United Kingdom; 3 Platelet Biology Group, National Heart and Lung Institute, Imperial College, London, United Kingdom; Maastricht University, Netherlands

## Abstract

**Background:**

Clinical use of selective inhibitors of cyclooxygenase (COX)-2 appears associated with increased risk of thrombotic events. This is often hypothesised to reflect reduction in anti-thrombotic prostanoids, notably PGI_2_, formed by COX-2 present within endothelial cells. However, whether COX-2 is actually expressed to any significant extent within endothelial cells is controversial. Here we have tested the effects of acute inhibition of COX on platelet reactivity using a functional *in vivo* approach in mice.

**Methodology/Principal Findings:**

A non-lethal model of platelet-driven thromboembolism in the mouse was used to assess the effects of aspirin (7 days orally as control) diclofenac (1 mg.kg^−1^, i.v.) and parecoxib (0.5 mg.kg^−1^, i.v.) on thrombus formation induced by collagen or the thromboxane (TX) A_2_-mimetic, U46619. The COX inhibitory profiles of the drugs were confirmed in mouse tissues *ex vivo*. Collagen and U46619 caused *in vivo* thrombus formation with the former, but not latter, sensitive to oral dosing with aspirin. Diclofenac inhibited COX-1 and COX-2 *ex vivo* and reduced thrombus formation in response to collagen, but not U46619. Parecoxib inhibited only COX-2 and had no effect upon thrombus formation caused by either agonist.

**Conclusions/Significance:**

Inhibition of COX-1 by diclofenac or aspirin reduced thrombus formation induced by collagen, which is partly dependent upon platelet-derived TXA_2_, but not that induced by U46619, which is independent of platelet TXA_2_. These results are consistent with the model demonstrating the effects of COX-1 inhibition in platelets, but provide no support for the hypothesis that acute inhibition of COX-2 in the circulation increases thrombosis.

## Introduction

It was first suggested over a decade ago that inhibitors of cyclooxygenase (COX)-2 might increase thrombotic risk [1], [2]. Support for this idea quickly followed in the results from clinical trials of selective COX-2 inhibitors. For example, in the Vioxx Gastrointestinal Outcomes Research (VIGOR) study, an increased rate of myocardial infarctions was reported in patients receiving the selective COX-2 inhibitor, rofecoxib, compared to the non-selective COX-1/COX-2 inhibitor, naproxen [3]. It has since become clear that almost all agents that inhibit COX-2, i.e. both selective COX-2 inhibitors and non-selective, non-steroidal anti-inflammatory drugs (NSAIDs), are associated with some pro-thrombotic tendency [4], [5], [6], [7], [8], [9], [10]. It is often hypothesised that this reflects inhibition of COX-2 in the vascular endothelium, and therefore reduced production of anti-thrombotic prostanoids, notably prostacyclin (PGI_2_). Despite this hypothesis there is remarkably little evidence from histochemical studies for the expression of COX-2 by healthy endothelial cells, where COX-1 appears to be the dominant isoform [7], [9], [11], [12], [13]. Indeed, it may be that other consequences of COX-2 inhibition, notably increases in fluid retention and blood pressure [6], [7], [9], [12], [14], provide better mechanistic explanations of the pro-thrombotic effects of drugs that inhibit COX-2.

Prostanoids are synthesised *de novo* without storage and generally have short half lives within the body [12], [15], [16]. As such, any contribution of COX-2-derived prostanoids to platelet reactivity should be sensitive to acute application of COX-2 inhibitors. Here we have tested this reasoning using the injectable, selective COX-2 inhibitor, parecoxib [17], in an established mouse model of *in vivo* thrombosis. For comparison and to confirm the role of platelet COX-1-derived thromboxane (TX) A_2_ in this model, we have also studied the effects of an injectable form of the non-selective NSAID, diclofenac, and chronic oral dosing with aspirin. Using this approach we find no evidence for an effect of acute COX-2 inhibition on thrombotic responses *in vivo*.

## Results

### Characterisation of thrombotic response

**Figure 1:**
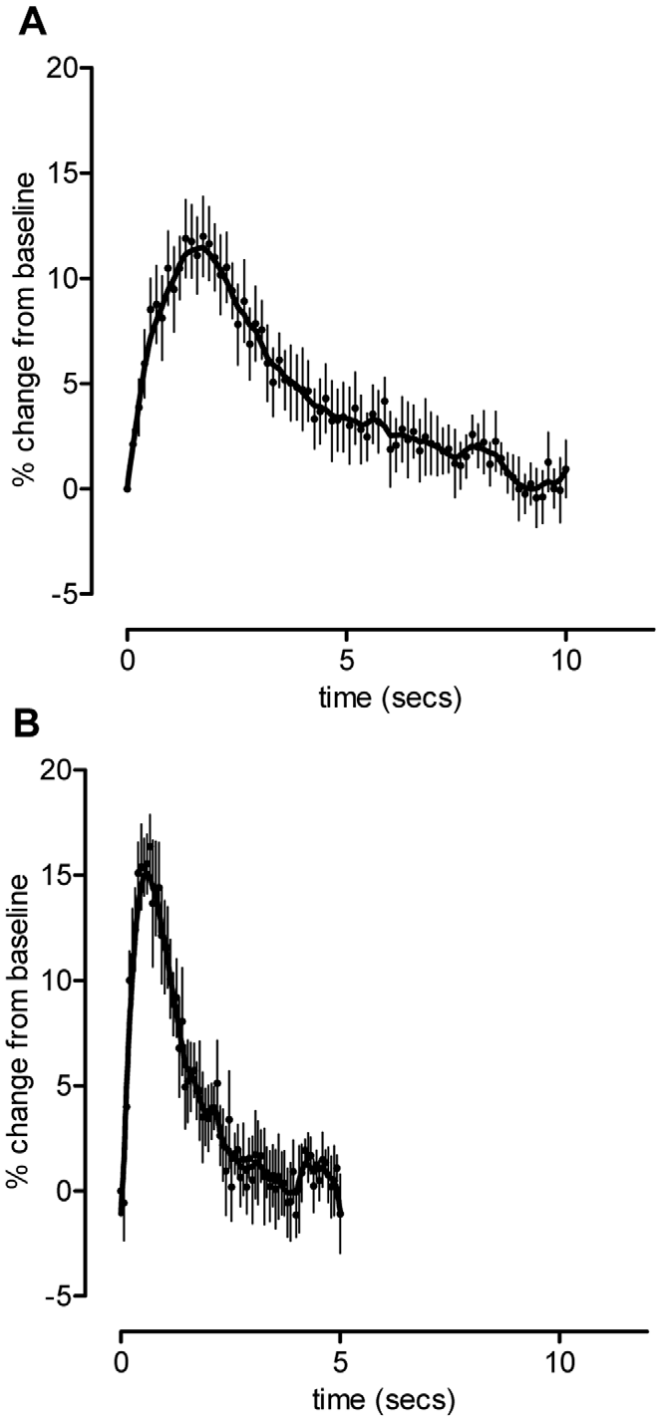
Representative pooled curves of platelet response. Radioactivity levels were recorded following administration of either collagen (50 µg.kg^−1^, i.v.; n = 7; upper panel) or U46619 (210 µg.kg^−1^, i.v.; n = 7; lower panel). Data is represented as % change from baseline (mean ± SEM) and plotted over time.

Injection of collagen (50 µg.kg^−1^, *i.v*) caused an increase in platelet accumulation in the lung that peaked around 100 seconds before gradually returning to baseline within 10 minutes (Figure 1). The response to the TXA_2_ mimetic, U46619 (210 µg.kg^−1^, *i.v*) was greater in magnitude than that to collagen but shorter lasting - the maximum was achieved after 40 seconds returning to baseline within 2–3 minutes.

### Effect of chronic aspirin dosing on COX-1 activity *ex vivo*

The production of TXA_2_ by platelets was reduced 87% (p<0.01) in blood taken from mice that had received 7 days administration of 100 mg.kg^−1^.day^−1^ p.o. aspirin, but not significantly altered in blood from mice receiving lower doses (all p>0.05). Based on these results the dose of 100 mg.kg^−1^.day^−1^ p.o. was chosen for studies using the *in vivo* thrombosis model.

### Effect of chronic aspirin dosing on thrombotic response

**Figure 2:**
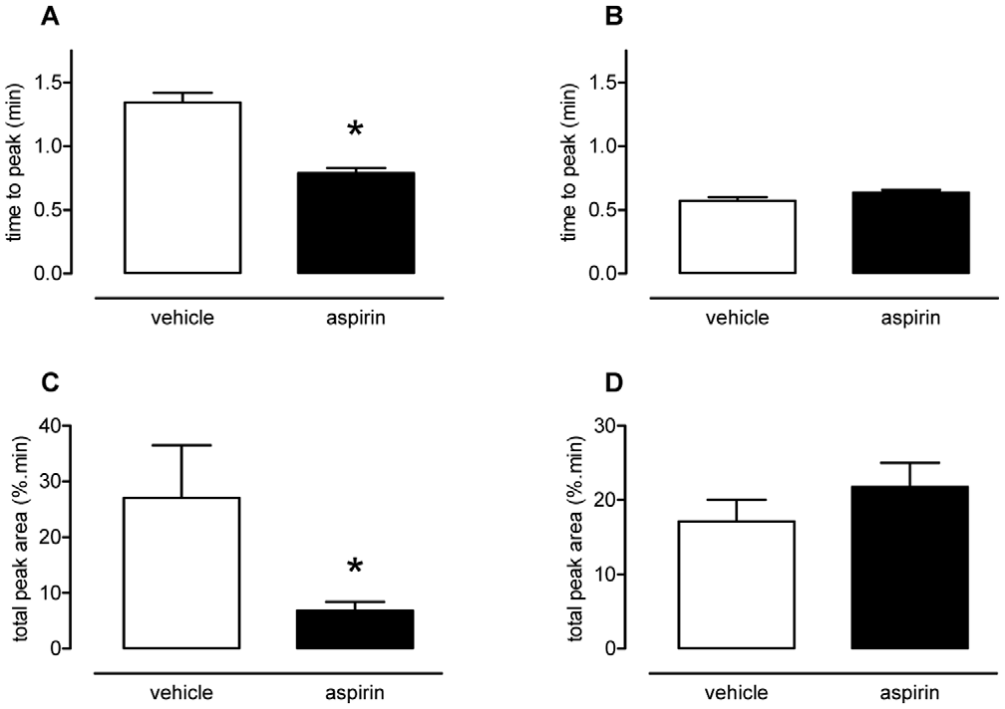
Effect of oral aspirin dosing on platelet response to collagen or U46619. From 6^th^ order polynomial regression analysis time to peak and total peak area were calculated for responses to collagen (50 µg.kg^−1^, i.v.; panels A and C) and U46619 (210 µg.kg^−1^, i.v.; panels B and D). In comparison to vehicle, aspirin (100 mg.kg^−1^.day^−1^ p.o. for 7 days) significantly reduced the time to peak (panel A) and total peak area (panel C). Aspirin had no effect upon responses to U46619 (panels B and D). Data presented as mean ± SEM, n = 6–7 per treatment group, *p<0.05 by one-way ANOVA and Dunnett's *post-hoc* test.

Treatment of mice with aspirin significantly reduced the time to peak (vehicle, 1.34±0.07 min; aspirin, 0.79±0.04 min; Figure 2A, p<0.05) and the total peak area (vehicle, 27.1±9.4%.min; aspirin, 6.9±1.6%.min; Figure 2C, p<0.05) of the response to collagen. Aspirin did not affect the response to U46619 (Figure 2B and D).

### Effect of acute diclofenac and parecoxib dosing on COX-1 and COX-2 activity *ex vivo*

**Figure 3:**
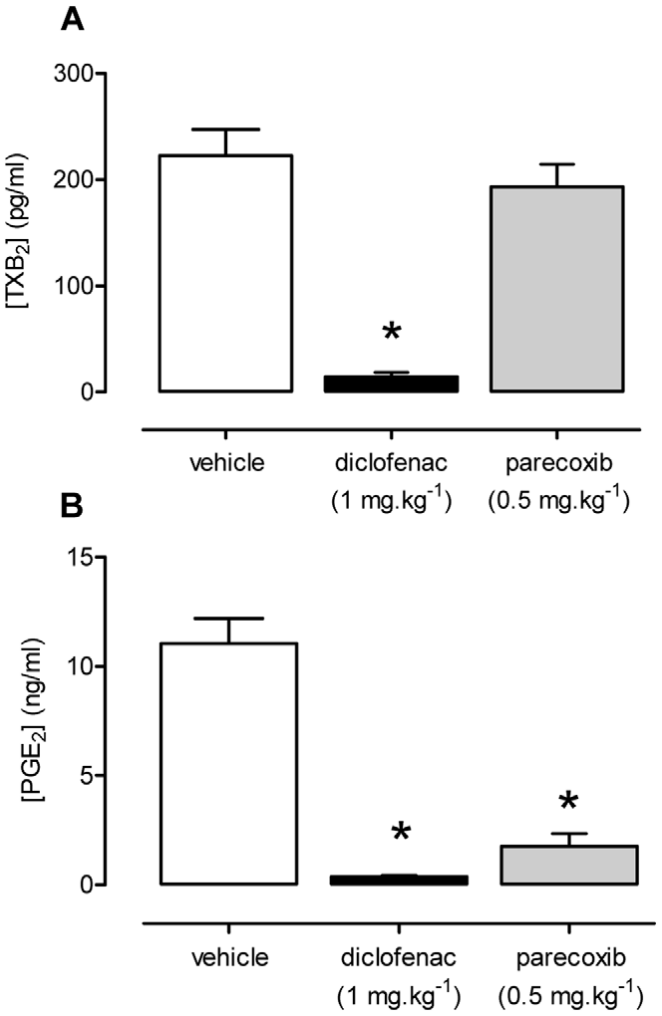
Effects of diclofenac and parecoxib treatment on COX-1 and COX-2 activity. Diclofenac (1 mg.kg^−1^, i.v.), but not parecoxib (0.5 mg.kg^−1^, i.v.), reduced the formation of TXA_2_ in Ca^2+^ ionophore-stimulated whole blood (panel A). Whole blood from mice treated with either diclofenac or parecoxib caused significant inhibition of PGE_2_ release from LPS-induced J774 macrophages (panel B). Data presented as mean ± SEM, *p<0.001 by one-way ANOVA and Dunnett's *post-hoc* test, n = 3.

In blood taken after acute administration of a standard clinical dose of diclofenac (1 mg.kg^−1^; i.v.) both COX-1-dependent production of TXA_2_ by platelets (Figure 3A) and the COX-2-dependent production of PGE_2_ by LPS-induced J774 macrophages was strongly inhibited (Figure 3B). In comparison only COX-2 activity was inhibited in blood taken after acute administration of parecoxib (0.5 mg.kg^−1^, i.v.; Figure 3A and B).

### Effect of diclofenac or parecoxib on thrombotic response to collagen or U46619

**Figure 4:**
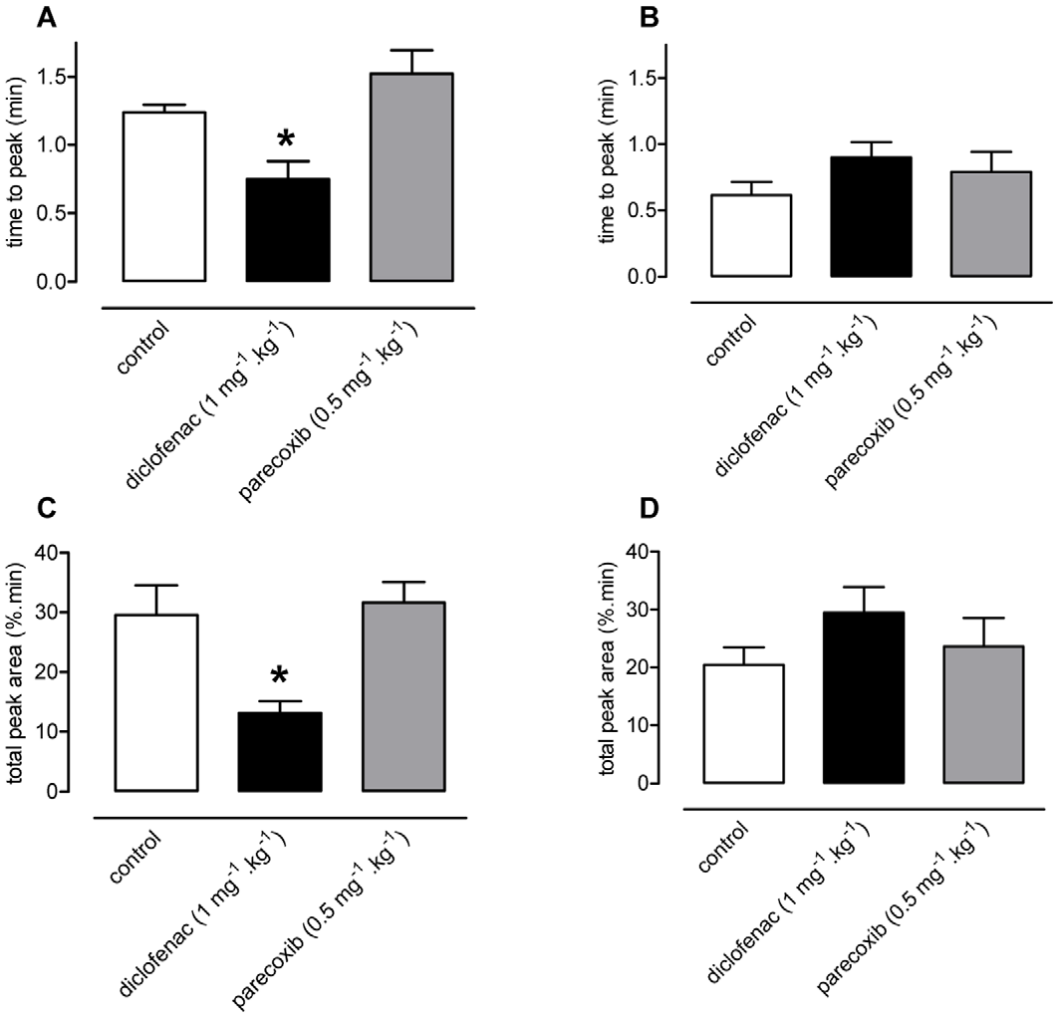
Effects of diclofenac and parecoxib treatment on collagen or U46619-induced platelet response. Diclofenac (1 mg.kg^−1^), but not parecoxib (0.5 mg.kg^−1^), significantly reduced the time to peak (panel A) and total peak area (panel C) of the thrombotic response to collagen. Neither diclofenac, nor parecoxib, significantly affected the thrombotic responses to U46619 (panels B and D). Data presented as mean ± SEM, n = 4–9 per treatment group, *p<0.05 by one-way ANOVA and Dunnett's *post-hoc* test.

Diclofenac produced similar effects on *in vivo* thrombosis to aspirin; namely a reduction in time to peak (control, 1.24±0.06 min; diclofenac, 0.75±0.13 min; Figure 4A, p<0.05) and a reduction in total peak area (control, 29.5±5.0%.min; diclofenac, 13.1±1.2%.min; Figure 4C, p<0.05). Parecoxib, in contrast, did not alter any parameter of the thrombotic response to collagen (Figure 4A and C). Neither diclofenac nor parecoxib significantly affected thrombosis induced by U46619 (Figure 4B and D).

## Discussion

The association of COX-2 inhibitors with increased risk of cardiovascular events has prompted a wide search for the causative mechanisms. A current leading hypothesis is that COX-2 is expressed in the endothelium and is responsible for the production of the vasodilating and anti-thrombotic prostanoids, particularly PGI_2_. This hypothesis remains controversial, however, as immunoreactive COX-2 is generally absent from healthy endothelium *in vivo* and *in vitro* whereas COX-1 is relatively abundant [7], [11], [12], [13]. Therefore we have sought to further our understanding of this area by functionally exploring the impact of a selective COX-2 inhibitor parecoxib on platelet reactivity, *in vivo*, in mice. We have found that, whilst this model could clearly reveal the well established anti-thrombotic effects of platelet COX-1 inhibition by chronic aspirin administration [18], [19] or acute diclofenac administration, it did not indicate any pro-thrombotic effect of selective COX-2 inhibition.

This study employed the modified pulmonary thromboembolism model described by Tymvios *et al* [20]. In this model, radioactively-labelled donor mouse platelets are injected into a recipient mouse and allowed to equilibrate, before administration of an agonist via the femoral vein. A subsequent increase of radiation, due to thrombi formation, can then measured in the pulmonary bed. As such, platelet reactivity can be determined where under the physiological influence of endogenous mediators [21], such as endothelium-derived prostanoids, an environment that is impossible to faithfully replicate either *in vitro* or *ex vivo*.

For this study, we chose two thrombotic agonists - collagen and the TXA_2_ mimetic, U46619. Collagen is known to be a key physiological activator of platelets [22], [23] and collagen-induced platelet aggregation is at least partly driven by platelet COX-1-derived TXA_2_, making this response sensitive to aspirin treatment [24], [25], [26], [27], [28]. U46619 mimics the effects of platelet-derived TXA_2_ but is not affected by inhibition of platelet COX-1 [24], [27], [28]. Moreover, *in vitro*, at least, both collagen- and U46619-induced platelet aggregations are sensitive to inhibition by PGI_2_ [29], [30], [31].

In order to confirm the model's suitability and sensitivity we first examined the effects of oral aspirin dosing, a well established anti-thrombotic treatment, on responses to collagen and U46619. It has previously been shown that the effective oral doses of aspirin in the mouse are substantially higher than those in humans [32]. Indeed, upon testing the ability of mouse platelets to synthesise TXA_2_, following 7 days of aspirin treatment, we found that a dose of 100 mg.kg^−1^day^−1^ was required in order to achieve significant reductions; approximately 100x that required in man [18], [19]. Regardless, at this dose, which inhibited platelet TXA_2_ production, *in vivo* thrombotic responses to collagen but not U46619 were impaired, thus validating the model and confirming the role of platelet TXA_2_ synthesis in it.

The roles and influences of COX-1 and COX-2 in collagen and U46619-induced thrombosis *in vivo* were determined by acute administration of the non-selective COX-1/COX-2 inhibitor, diclofenac, and the selective COX-2 inhibitor, parecoxib. In these acute studies we deliberately used intravenous application of parecoxib and diclofenac to produce therapeutically relevant levels of drugs within the circulation. Data from our studies using orally administered aspirin demonstrated the common observation that to produce effects upon the target enzymes NSAIDs need to be given orally to mice at doses very much higher than those used in humans. In a study such as the one presented here, where drugs are being used for their pharmacological selectivities, this clearly presents an important problem; i.e. are drugs still selective at these much higher doses. Because prostanoids are very short lived in the circulation and their enzymatic sources are still a matter of debate, measurement of circulating prostanoid levels is of little assistance in determining drug activities. We therefore took blood from the animals following injection of drugs or vehicle and tested the levels of active drugs by bioassay in defined systems; i.e. employing COX-1-dependent formation of TXA_2_ in platelets, and COX-2-dependent formation of PGE_2_ in mouse monocytes. We have published such approaches previously [33], [34], [35]. These studies confirmed that at the dose used, diclofenac was present in the blood at a concentration that inhibited both COX-1 and COX-2; parecoxib was present in the blood at a concentration that inhibited COX-2 but not COX-1. Diclofenac mimicked the effects of aspirin – reducing the thrombotic response to collagen without altering that to U46619. This effect on collagen-induced thrombosis is consistent with the observed ability of diclofenac to inhibit platelet TXA_2_ formation in our experiments, and with the effects of diclofenac in healthy humans [36], [37]. Interestingly, despite the administration of a dose of diclofenac that strongly inhibits COX-2 *ex vivo*, no increase in U46619-induced thrombosis was seen. This suggests that U46619-induced thrombosis is not acutely suppressed by COX-2-dependent release of prostanoids from the vascular wall. More compellingly, parecoxib [17], at a dose observed to inhibit COX-2 with little effect on COX-1, also did not increase the thrombotic responses to either collagen or U46619. This, again, suggests that COX-2-dependent PGI_2_ formation does not suppress platelet reactivity in this model.

Taken together, these findings provide no support for the hypothesis that inhibition of COX-2 in the vascular wall acutely alters the local haemostatic environment. In particular, any contribution of COX-2 to the formation of anti-thrombotic prostanoids should have been strongly diminished by the doses of parecoxib or diclofenac used in this study, yet no increase in thrombosis was noted with either treatment. Indeed, there was a more noticeable, though non-significant, trend to increased U46619-induced thrombosis in the presence of diclofenac than in the presence of parecoxib, which might conceivably reflect the role of COX-1 in the production of anti-thrombotic prostanoids.

In conclusion, we demonstrate here that acute administration of the selective COX-2 inhibitor, parecoxib, has no detectable effects in this *in vivo* model of platelet activation and thrombosis. This result would appear consistent with the common finding that COX-1 rather than COX-2 is the predominant COX isoform present in normal vasculature and provides no support for the concept of COX-2-dependent anti-thrombotic prostanoid production by the healthy blood vessel wall. Of course, the relative contributions of COX-1 and COX-2 to prostanoid production will differ in blood vessels with atherosclerotic disease and elevated expression of COX-2 [5], [7], conditions that may be more relevant to the patient groups that use chronic NSAIDs.

## Materials and Methods

### Ethics statement

All procedures described in this study were subject to Home Office approval (PPL 70–7013) under “The Animals (Scientific Procedures) Act 1986” and local approval from Imperial College London Local Ethical Review Panel.

### Mice

Male BALB/c mice of 7–8 weeks old and 20–25 g (Harlan, UK) were received a minimum of 7 days before the commencement of experiments. They were housed on a 12 hour light-dark cycle, at a temperature of 22–24°C with access to water and food *ad libitum*.

### Aspirin, diclofenac or parecoxib administration

For aspirin dosing, mice received daily oral doses of 1–300 mg.kg^−1^.day^−1^ via gavage for 7 days. Aspirin (Sigma, UK) was finely ground using a mortar and pestle before weighing and suspension in a 4% tragacanth solution (Sigma, UK; in water). For diclofenac and parecoxib dosing, mice received injectable forms of diclofenac (1 mg.kg^−1^; Voltarol®, Geigy), parecoxib (0.5 mg.kg^−1^; Dynastat®, Pfizer) or vehicle by tail vein injection.

### *Ex vivo* COX-1 and COX-2 activity assays

30 minutes after dosing as described above, mice subject to each treatment were killed with CO_2_ and blood collected from the inferior vena cava into heparin (10U.ml^−1^ final concentration; CP Pharmaceuticals Ltd). To determine the level of COX-1 inhibitory activity following drug administration, 100 µl of each blood sample was incubated with Ca^2+^ ionophore A23187 (50 µM; Sigma) for 30 minutes before termination of COX activity by addition of diclofenac (1 mM; Sigma) and separation of plasma by centrifugation. TXA_2_ production was measured by enzyme immunoassay (Cayman Chemical, USA) for its stable breakdown product, TXB_2_, as an index of platelet COX-1 activity.

To determine the level of COX-2 inhibitory activity following drug administration, 100 µl of each blood sample was applied to J774 murine macrophages that had been incubated with LPS (10 ug.ml^−1^; from E. coli 0111:B4; Sigma) for 24 hours to induce COX-2. After 30 minutes equilibration period, cells were stimulated by incubation with Ca^2+^ ionophore A23187 (50 µM; Sigma, UK) for a further 30 minutes. COX activity was terminated by addition of diclofenac (1 mM; Sigma, UK), and plasma separated by centrifugation. Prostaglandin E_2_ production was determined by a homogeneous time resolved fluorescence-based immunoassay (Cisbio, France), as an index of J774 COX-2 activity.

### Platelet isolation and radio-labelling

Donor mice were anaesthetised with 2.5 mg.kg^−1^ urethane (as 25% solution, i.p; Sigma, UK). Blood was collected from terminally anaesthetised donor mice by cardiac puncture into acidified citrate-dextrose solution. Platelet rich plasma (PRP) was obtained by two-step centrifugation (30 *g*, 3 mins) to remove extraneous erythrocytes and white blood cells. PRP was supplemented with an equal volume of Ca^2+^-free Tyrode's solution (CFT: 125 mM glucose, 2.5 mM KCl, 0.4 mM NaH_2_PO_4_, 5 mM glucose, 11 mM NaHCO_3_, 6.8 mM trisodium citrate, 3.8 mM citric acid) containing prostaglandin E_1_ and centrifuged to produce a platelet pellet. The platelet pellet was washed carefully with CFT, re-suspended with 1.8 MBq ^111^Indium oxine and incubated at room temperature for 5 minutes. Platelets were re-pelleted by a final centrifugation, washed with CFT and re-suspended in 50 µl CFT per mouse.

### *In vivo* thrombosis model

The murine *in vivo* thrombosis model was conducted as previously published [20]. Briefly, recipient mice were anaesthetised as above and infused via a tail vein with radio-labelled donor platelets, prepared as above. Animals were then allowed to equilibrate for 20 minutes before platelet agonists, collagen (50 µg.kg^−1^; Nycomed, Germany) or U46619 (210 µg.kg^−1^; Cayman Chemical, USA) were administered via an exposed femoral vein. Platelet responses were then determined as increases in platelet-associated counts in the pulmonary vascular bed associated with the platelet agonists. Data was collected via 1 cm SPEAR (Single Point Extended Area Radiation) detectors (eV Products, PA, USA) fixed over the pulmonary vascular bed and recorded on a UCS-20 spectrometer (Spectrum Techniques, Oak Ridge, TN, USA) using custom made software (Mumed Systems, London, UK).

### Statistical analysis

Results are presented as mean ± SEM and values of p<0.05 were considered to be significant. Radioactivity counts were converted into % change from baseline and plotted over time. Traces were fitting to 6^th^ order polynomial regression curves to allow the calculation of time to peak and total peak area. All analysis was performed using Prism 4.0 software (GraphPad Software, USA).
